# Unusual Presentation of Streptococcus uberis Bacteremia in a Complex Patient: A Case Report

**DOI:** 10.7759/cureus.82157

**Published:** 2025-04-12

**Authors:** Jinalben Chaudhari, Priyasheelta Nand

**Affiliations:** 1 Internal Medicine, St. Joseph's Medical Center, Stockton, USA; 2 Internal Medicine/Infectious Diseases, St. Joseph's Medical Center, Stockton, USA

**Keywords:** kaposi sarcoma, sirolimus, streptococcus uberis bacteremia, tricuspid valve endocarditis necessitating valve replacement, vancomycin

## Abstract

*Streptococcus uberis (S. uberis)*, primarily recognized as a pathogen in bovine mastitis, rarely causes infections in humans. Here, we present a case of *S. uberis* bacteremia in a 58-year-old male patient with multiple comorbidities, including end-stage renal disease (ESRD) on hemodialysis, type 2 diabetes mellitus, and a history of tricuspid valve endocarditis.

The patient presented with generalized weakness, fatigue, and diarrhea, alongside intermittent chills and elevated heart rate during hemodialysis. Initial evaluation revealed severe sepsis with elevated lactic acidosis and abnormal cardiac biomarkers. Blood cultures confirmed *S. uberis* bacteremia, prompting empirical treatment with vancomycin and cefepime adjusted for dialysis clearance. A transthoracic echocardiogram (TTE) was performed to evaluate for infective endocarditis.

The patient responded well to antibiotic therapy, with the resolution of fever and inflammatory markers. Subsequent investigations, including a transesophageal echocardiogram (TEE), did not reveal valvular vegetations. The patient completed six weeks of vancomycin therapy and was advised on outpatient follow-up for further management.

This case highlights the diagnostic challenges and therapeutic considerations in managing *S. uberis* bacteremia in immunocompromised individuals. Despite the bacterium's primary association with dairy cattle, our patient's lack of direct contact underscores the potential for zoonotic transmission through environmental exposures. Effective management included prompt initiation of appropriate antibiotics guided by susceptibility testing and a comprehensive evaluation for infective endocarditis. Further research is needed to elucidate optimal management strategies for *S. uberis* infections in vulnerable patient populations.

## Introduction

*Streptococcus uberis* *(S. uberis)*, a gram-positive bacterium, is primarily recognized as a causative agent of bovine mastitis, leading to significant economic losses in the dairy industry due to reduced milk production and quality [1]. Although it is commonly found in the environment, particularly in straw, bedding, and soil, human infections with *S. uberis* are rare and not well-characterized in the medical literature. Recent case reports have documented its ability to cause opportunistic infections in humans, particularly in immunocompromised individuals, where it has been implicated in bacteremia, endocarditis, pneumonia, and urinary tract infections [2,3]. However, due to its rarity in human pathology, there remains a gap in understanding its true pathogenicity, transmission, and optimal management in human hosts.

The zoonotic potential of *S. uberis* has been suggested by multiple case reports, with documented infections in individuals with direct or indirect exposure to livestock and dairy products [4]. In dairy cattle, the bacterium primarily gains entry into the mammary gland through the teat canal, leading to mastitis characterized by swelling, pain, and abnormalities in milk composition [1]. Given its ability to thrive in various environmental conditions, there is a theoretical risk of transmission to humans, especially those in close contact with contaminated materials. However, in some reported cases, including our patient, no direct animal exposure was identified, suggesting that alternative transmission routes, such as environmental contamination or transient colonization, may play a role in human infections.

Immunocompromised patients, particularly those with end-stage renal disease (ESRD) requiring hemodialysis, prolonged immunosuppressive therapy, or prosthetic valve replacements, are at heightened risk for opportunistic infections [5]. These patients often exhibit impaired immune responses, making them more susceptible to infections by uncommon pathogens. Given the rarity of *S. uberis* in human disease, clinicians may overlook its clinical significance, leading to potential delays in diagnosis and treatment. This case highlights the importance of considering unusual pathogens in the differential diagnosis of bacteremia in immunocompromised individuals, emphasizing the role of advanced microbiological techniques, timely antimicrobial therapy, and a multidisciplinary approach to optimizing patient outcomes.

## Case presentation

The patient is a 58-year-old male patient with a complex medical history including ESRD on hemodialysis, type 2 diabetes mellitus (T2DM), hypertension (HTN), duodenal carcinoid tumor, tricuspid valve endocarditis necessitating valve replacement, failed right kidney transplant managed with prednisone and sirolimus, Kaposi sarcoma treated with chemotherapy, and osteomyelitis. He has received ongoing care, including his renal transplant and current hemodialysis via a right upper extremity (RUE) fistula.

**Figure 1:**
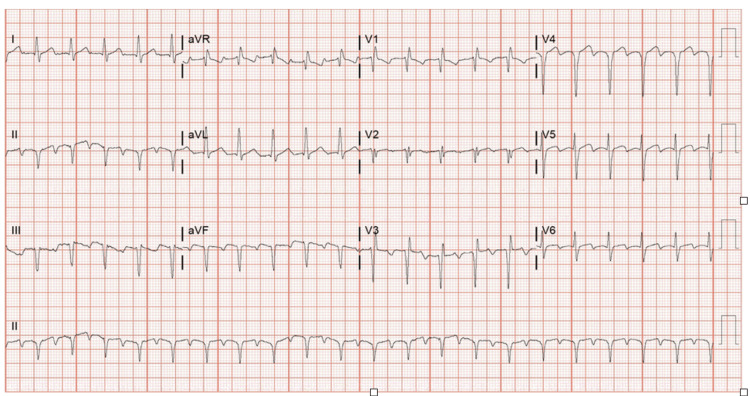
EKG showing ectopic atrial tachycardia with a heart rate of 126 bpm, incomplete right bundle branch block, and biphasic T waves in leads V3-V6, without ST segment changes or previous T wave abnormalities

The patient was presented to the emergency department due to generalized weakness, fatigue, diarrhea for four days, intermittent chills, and a history of feeling tired with elevated heart rate noted during a recent dialysis session. On admission, he was tachycardic with an abnormal EKG, as seen in Figure 1, suggestive of ectopic atrial tachycardia and incomplete right bundle branch block. Initial evaluation revealed severe sepsis with lactic acidosis, prompting further investigation.

Physical examination revealed tenderness in the right lower quadrant (RLQ), attributed to chronic pain following his kidney transplant. Laboratory tests on admission are shown in Table 1.

**Table 1:** Labs on admission BNP: B-type natriuretic peptide; BUN: blood urea nitrogen

Test	Patient’s value	Normal range
BNP	1639 pg/mL	<100 pg/mL
BUN	23.6 mg/dL	7–20 mg/dL
Creatinine	4.5 mg/dL	0.6–1.2 mg/dL
Glucose	160 mg/dL	70–140 mg/dL (postprandial)
Hemoglobin (Hb)	11.3 g/dL	13.0–17.0 g/dL (M) / 12.0–15.0 g/dL (F)
Lactic acid	5.9 g/dL	0.5–2.2 g/dL
Sodium	136 mmol/L	135–145 mmol/L
WBC	10.5 × 10⁹/L	4.0–11.0 × 10⁹/L

**Figure 2:**
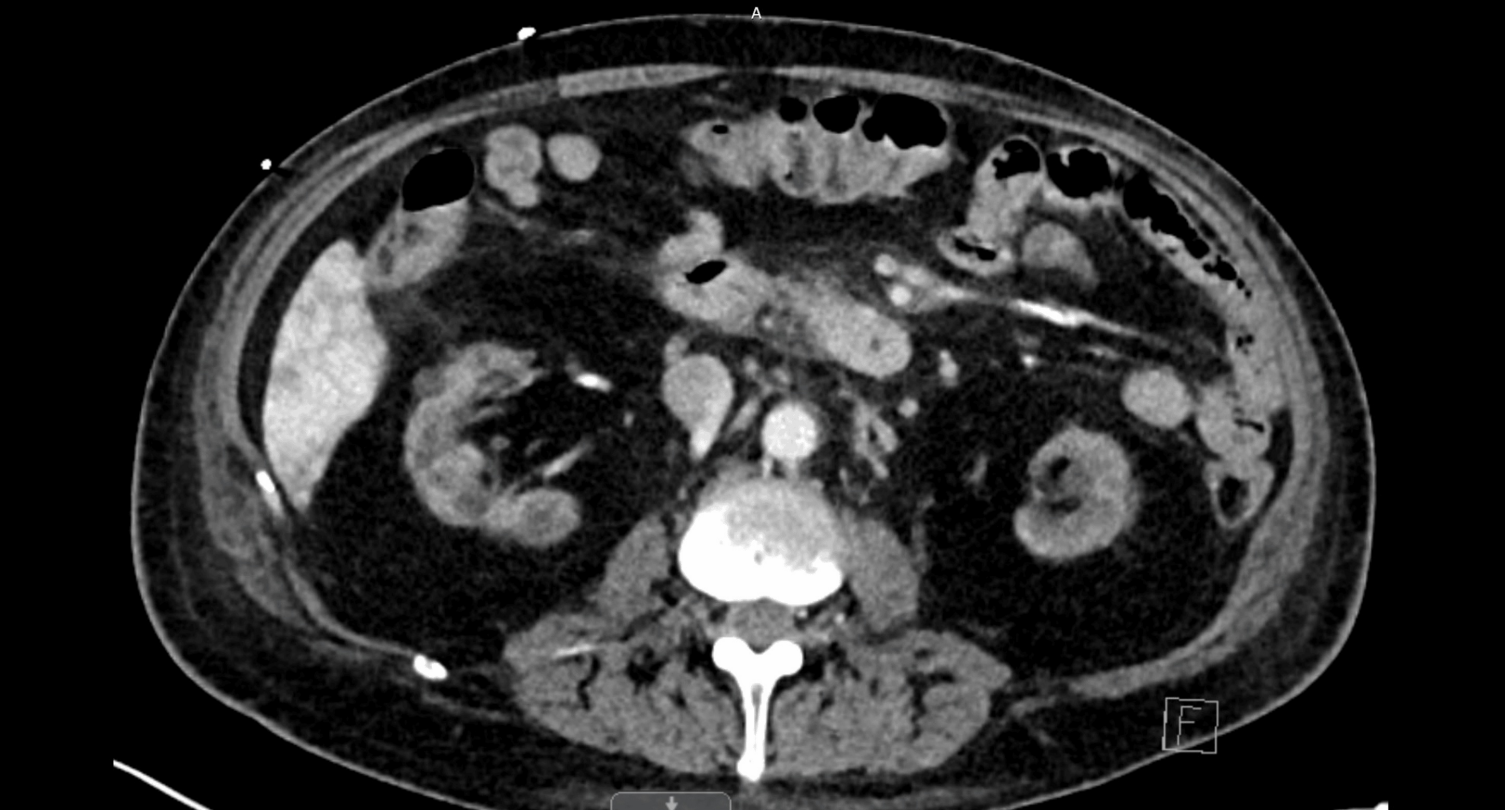
Mildly heterogeneous enhancement of the renal transplant parenchyma with mildly increased perirenal stranding suggesting pyelonephritis of the right iliac fossa

A CT scan revealed changes suggestive of mild right pyelonephritis, as seen in Figure 2. Blood cultures collected on admission grew *S. uberis* in all four bottles. Given the bacteremia and suspicion of an underlying source, the patient was started on empiric treatment with vancomycin and cefepime.

Infectious disease specialists were consulted for guidance on managing *S. uberis* bacteremia. Given the patient's history of immunosuppression and prosthetic valve, concerns for endocarditis were raised. A transthoracic echocardiogram (TTE) was performed to evaluate for valvular abnormalities and vegetations.

The patient was stabilized on antibiotic therapy with vancomycin adjusted for dialysis clearance (1 gram per dialysis session), alongside cefepime based on susceptibility results as listed in Table 2.

**Table 2:** Antimicrobial susceptibility for Streptococcus uberis S (susceptible): The isolate is inhibited by the antibiotic at standard therapeutic doses, indicating effective treatment; I (intermediate): The isolate has reduced susceptibility; higher doses or specific site concentrations may be needed for effectiveness; R (resistant): The isolate is not effectively inhibited by the antibiotic, making treatment unlikely to succeed; MINT (minimal inhibitory non-toxic concentration): The lowest concentration of an antibiotic that inhibits bacterial growth without causing toxicity; MDIL (minimum detectable inhibitory level): The lowest concentration at which bacterial growth is visibly inhibited

Drug	MINT	MDIL
Ampicillin	S	0.25
Cefepime	S	1
Cefotaxime	I	2
Ceftriaxone	I	2
Clindamycin	S	<=0.06
Erythromycin	S	<=0.06
Penicillin	S	0.12
Vancomycin	S	1

Given the patient’s hemodialysis dependency and use of nephrotoxic agents like vancomycin, renal function was closely monitored through serial serum creatinine, vancomycin trough levels, and dialysis-adjusted dosing per institutional protocol. Dose adjustments were made based on pharmacokinetic monitoring and infectious disease consultation. While *S. uberis* is a rare pathogen in humans and lacks specific treatment guidelines, our antibiotic choices were guided by general principles for gram-positive bacteremia management in immunocompromised hosts. According to Baddour et al., for the management of bacteremia and endocarditis caused by streptococci, empiric therapy with vancomycin and cefepime is appropriate in immunocompromised patients pending culture and susceptibility results [5]. Vancomycin was continued in our patient, given its coverage and pending susceptibility data, which ultimately demonstrated sensitivity to beta-lactams as well. For immunocompromised individuals, the recommended duration of therapy for uncomplicated bacteremia is generally 14 days, while cases with suspected or confirmed endocarditis or prosthetic valve involvement typically require a longer course, often four to six weeks. On this regimen, his clinical condition improved with resolution of fever and improvement in inflammatory markers. Thus, six weeks of vancomycin therapy were planned, followed by outpatient follow-up with infectious disease specialists for further management and monitoring.

## Discussion

*S. uberis*, a gram-positive bacterium primarily associated with mastitis in dairy cows, is recognized for its ability to opportunistically invade the bovine udder, leading to inflammation and significant economic losses due to compromised milk production and quality [1]. Abd El-Aziz et al. elaborate on its pathogenesis in cows, where *S. uberis* typically gains entry into the mammary gland through the teat canal during milking or via environmental contamination of bedding and equipment [1]. Once inside, the bacterium adheres to and colonizes mammary epithelial cells, evading host immune responses and producing toxins and enzymes that contribute to tissue damage and inflammation. Clinical signs of *S. uberis* mastitis in cows include swollen and painful udders, along with abnormalities in milk appearance, such as clots or flakes [1].

In rare instances, *S. uberis* has been implicated in human infections, particularly among immunocompromised individuals or those with underlying health conditions [2]. Our patient, with a history of ESRD, immunosuppression from chronic prednisone and sirolimus therapy, and a complex medical history including a prosthetic tricuspid valve derived from bovine pericardium, represents a vulnerable population at heightened risk for opportunistic infections.

Valentiny et al. provide a comprehensive review of documented cases of *S. uberis* infections in humans, revealing a spectrum of clinical manifestations ranging from urinary tract infections to more severe conditions such as pneumonia, abscess formations, endocarditis, gonarthritis, and postoperative endophthalmitis [2]. This diversity in clinical presentation highlights the bacterium's adaptability in causing infections across various demographic profiles despite its rarity in human hosts.

The association between *S. uberis* infections and frequent contact with cows and milk, as observed by Gülen et al., further supports its zoonotic potential [3]. In rural settings, such exposures may facilitate transmission to humans, leading to localized infections like urinary tract infections [3,4]. Although our patient denied recent direct contact with dairy animals, his occupational history on a farm in the past suggests a potential reservoir for exposure. Management typically involves antibiotic therapy tailored to the bacterium's susceptibility profile, reflecting the importance of accurate identification and targeted treatment strategies.

In systemic infections such as bacteremia and pneumonia reported by Sarkar and Gould, effective management with intravenous antibiotics, including penicillin and broader-spectrum agents as warranted by susceptibility testing, has been pivotal in achieving clinical recovery [6]. Similarly, cases of abscess formation or arthritis following trauma or surgery underscore the need for combined surgical drainage and antimicrobial therapy to resolve localized infections [7].

Vigilant diagnostic approaches and timely therapeutic interventions are crucial in managing these potentially life-threatening infections.

This underscores the importance of occupational health measures and heightened awareness among healthcare providers for effective management of infections associated with environmental exposures.

In our case, the detection of *S. uberis* in all blood culture bottles, despite initial clinical skepticism regarding its significance, prompted a thorough microbiological evaluation and confirmation. The decision-making process was influenced by the bacterium's propensity for misidentification, as traditional biochemical phenotyping methods may not reliably distinguish it from closely related species such as *Enterococcus faecium*, as highlighted in the literature, necessitating advanced genotypic techniques like polymerase chain reaction (PCR) and sequencing for accurate diagnosis [8,9]. Our patient's management involved empirical antibiotic therapy with vancomycin and cefepime upon detection of *S. uberis* bacteremia (Table 2). This regimen was initiated promptly due to the organism's potential to cause severe systemic infections, guided by microbiological confirmation and susceptibility testing.

Given the patient's history of prosthetic valve and ongoing immunosuppression, concerns for infective endocarditis were raised, prompting a comprehensive evaluation, including transthoracic echocardiography (TTE) to assess for valvular abnormalities or vegetations. TTE initially did not reveal the presence of valvular vegetations, so a transesophageal echocardiogram (TEE) was performed and revealed no vegetations either. Given his history of tricuspid valve endocarditis with the presence of a prosthetic valve and a no clear source of his bacteremia, he was recommended to undergo six weeks of vancomycin therapy for treatment of possible valvular infection. In light of the suspected complicated bacteremia, the patient's long-term immunosuppression regimen, including prednisone and sirolimus, was reconsidered to reduce the risk of further opportunistic infections. Sirolimus was temporarily withheld during antibiotic therapy, and a recommendation to reevaluate long-term immunosuppression.

## Conclusions

This case highlights the diagnostic and therapeutic challenges associated with *S. uberis* infections in immunocompromised individuals. Due to its rarity in human infections and potential for misidentification, timely recognition and appropriate treatment are essential to prevent severe complications. Clinicians should maintain a high index of suspicion for unusual pathogens in immunocompromised hosts, particularly those with prosthetic devices or chronic immunosuppression. Accurate identification of *S. uberis* requires advanced microbiological techniques, including molecular assays and sequencing, to distinguish it from phenotypically similar organisms. Empirical antibiotic therapy should be guided by susceptibility testing to ensure optimal treatment outcomes. Additionally, this case underscores the importance of a multidisciplinary approach involving infectious disease specialists, microbiologists, and public health authorities to enhance diagnostic accuracy and management strategies.

Further research into the epidemiology, transmission, and antimicrobial resistance patterns of *S. uberis* in human infections is warranted. Increased surveillance and reporting of cases will provide valuable insights into its clinical significance, helping to refine preventive measures and treatment protocols. As zoonotic and environmental pathogens continue to emerge as potential infectious threats, a broader understanding of their role in human disease will be critical in improving patient outcomes.
